# Toward a decade of ocean science for sustainable development through acoustic animal tracking

**DOI:** 10.1111/gcb.16343

**Published:** 2022-08-05

**Authors:** Josep Alós, Kim Aarestrup, David Abecasis, Pedro Afonso, Alexandre Alonso‐Fernandez, Eneko Aspillaga, Margarida Barcelo‐Serra, Jonathan Bolland, Miguel Cabanellas‐Reboredo, Robert Lennox, Ross McGill, Aytaç Özgül, Jan Reubens, David Villegas‐Ríos

**Affiliations:** ^1^ Instituto Mediterráneo de Estudios Avanzados, IMEDEA (CSIC‐UIB) Esporles Spain; ^2^ Section for Freshwater Fisheries and Ecology National Institute of Aquatic Resources, Technical University of Denmark Silkeborg Denmark; ^3^ Center of Marine Sciences Universidade do Algarve (CCMAR) Faro Portugal; ^4^ Institute of Marine Research (IMAR/Okeanos), University of the Azores Horta Portugal; ^5^ Instituto de Investigaciones Marinas (IIM), CSIC Vigo Spain; ^6^ Hull International Fisheries Institute University of Hull Hull UK; ^7^ National Center Spanish Institute of Oceanography, CSIC Balearic Islands CO Spain; ^8^ NORCE Norwegian Research Center AS Bergen Norway; ^9^ Norwegian Institute for Nature Research Trondheim Norway; ^10^ Loughs Agency Derry/Londonderry UK; ^11^ Ege University Faculty of Fisheries Izmir Turkey; ^12^ Flanders Marine Institute Ostend Belgium

**Keywords:** acoustic tracking, climate change, fisheries, marine pollution, movement, networks, ocean monitoring, sustainable development, telemetry

## Abstract

The ocean is a key component of the Earth's dynamics, providing a great variety of ecosystem services to humans. Yet, human activities are globally changing its structure and major components, including marine biodiversity. In this context, the United Nations has proclaimed a Decade of Ocean Science for Sustainable Development to tackle the scientific challenges necessary for a sustainable use of the ocean by means of the Sustainable Development Goal 14 (SDG14). Here, we review how Acoustic animal Tracking, a widely distributed methodology of tracking marine biodiversity with electronic devices, can provide a roadmap for implementing the major Actions to achieve the SDG14. We show that acoustic tracking can be used to reduce and monitor the effects of marine pollution including noise, light, and plastic pollution. Acoustic tracking can be effectively used to monitor the responses of marine biodiversity to human‐made infrastructures and habitat restoration, as well as to determine the effects of hypoxia, ocean warming, and acidification. Acoustic tracking has been historically used to inform fisheries management, the design of marine protected areas, and the detection of essential habitats, rendering this technique particularly attractive to achieve the sustainable fishing and spatial protection target goals of the SDG14. Finally, acoustic tracking can contribute to end illegal, unreported, and unregulated fishing by providing tools to monitor marine biodiversity against poachers and promote the development of Small Islands Developing States and developing countries. To fully benefit from acoustic tracking supporting the SDG14 Targets, trans‐boundary collaborative efforts through tracking networks are required to promote ocean information sharing and ocean literacy. We therefore propose acoustic tracking and tracking networks as relevant contributors to tackle the scientific challenges that are necessary for a sustainable use of the ocean promoted by the United Nations.

## Graphical abstract

The United Nations has proclaimed a Decade of Ocean Science for Sustainable Development. Here, we review how Acoustic animal Tracking (AT), a widely distributed methodology of tracking marine biodiversity with electronic devices, can provide a roadmap for implementing the major Actions to achieve the Sustainable Development Goals of life below water. This review provides a list of specific examples in how AT can help reaching most Targets by providing cutting‐edge scientific data.

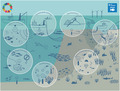

## 1 INTRODUCTION

The ocean is a key component of the Earth's dynamics and provides a great variety of ecosystem services (Barbier, 2017). Oceans and seas produce up to 16% of the animal protein used for human consumption and provides to approximately 3.3 billion people with almost 20% of their average per capita intake of animal protein (Duarte et al., 2009; Edwards et al., 2019; FAO, 2021). It is projected that oceans could generate food for almost 10 billion people by 2050 if managed in a sustainable way (Costello et al., 2020). However, human activity is drastically changing the structure and functioning of oceans, generating the ethical obligation to rebuild marine biodiversity and preserve the many benefits that society receives from a healthy ocean (Duarte et al., 2020).

**FIGURE 1:**
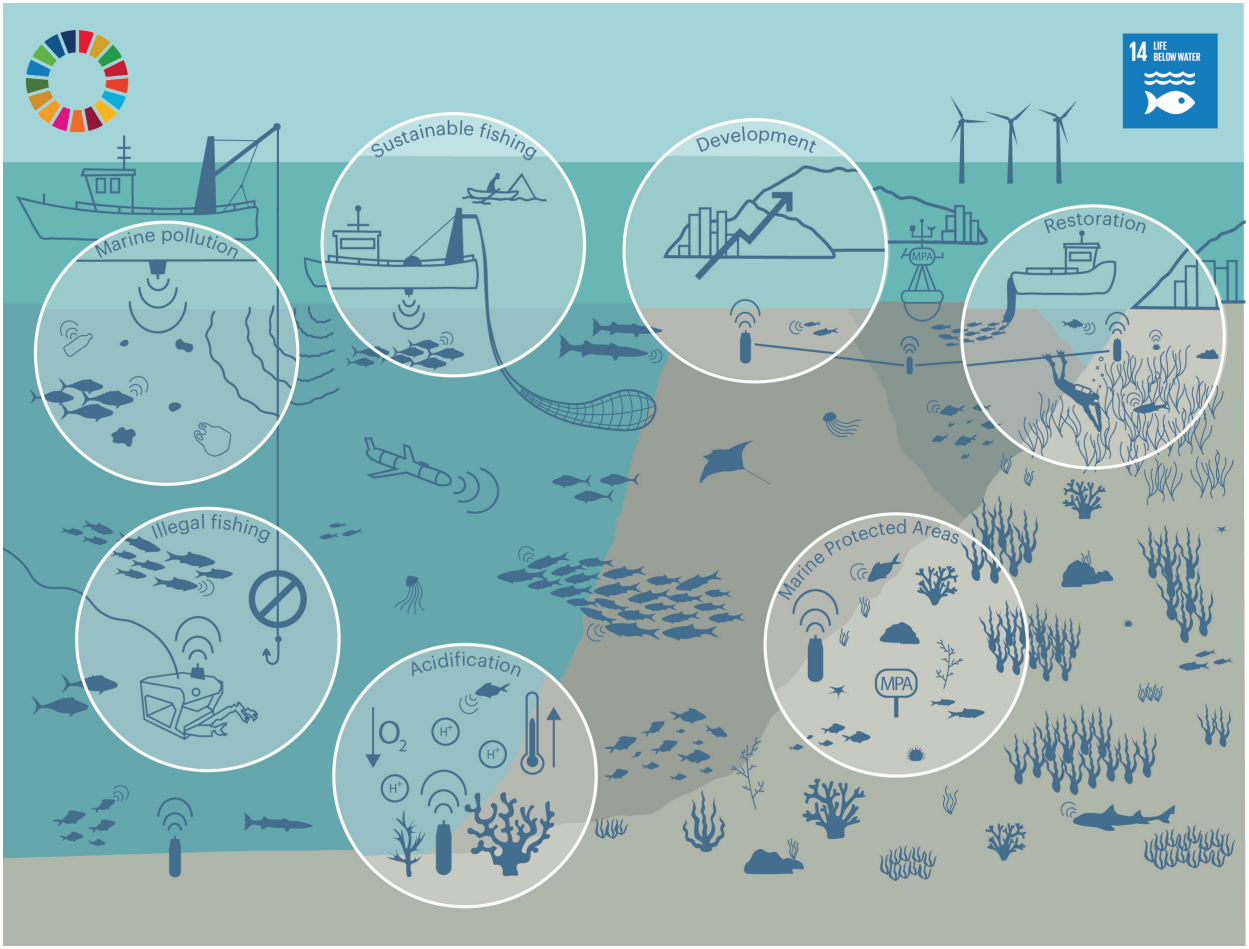
Toward a Decade of Ocean Science for Sustainable Development of Marine Biodiversity Using Acoustic animal Tracking (AT). The SDG14 identifies ten targets to create action to conserve and sustainably use the ocean: Target 14.1 reduce marine pollution; Target 14.2 protect and restore ecosystems; Target 14.3 reduce ocean acidification; Target 14.4 conserve coastal and marine areas; Target 14.5 sustainable fisheries; 14.6 end subsidies contributing to overfishing, illegal, unreported, and unregulated fishing; Target 14.7 increase the economic benefits to Small Island Developing States and least developed countries; Target 14.A increase scientific knowledge, research and technology for ocean health; Target 14.B support small‐scale fishers; and Target 14.C implement and enforce international sea law. This review provides a list of specific examples in how AT can help reaching these targets by providing cutting‐edge scientific data.

In this context, the United Nations has promoted the “Decade of Ocean Sciences for Sustainable Development (2021–2030)” (UN, 2015). This initiative is mainly focused on tackling the scientific objectives that are necessary for a sustainable use of natural resources (summarized in Ryabinin et al., 2019). Within this decade, the UN expects to encourage the scientific community, managers, non‐governmental organizations (NGOs), policy‐makers, as well as the general public to move beyond “business as usual” and aspire to a real social and economic change (Claudet et al., 2020), based on decision making informed by scientific criteria (Pendleton et al., 2020). The objectives to be tackled in the Decade of Ocean Sciences for Sustainable Development are described as the UN Sustainable Development Goals (SDGs), specifically within the SDG14, which refers to Life Below Water (https://sustainabledevelopment.un.org/sdg14). The Sustainable Development Goal SDG14 aims to preserve marine biodiversity and shift toward the sustainable use of oceans, seas and marine resources by following specific targets driven by scientifically informed managerial decisions (Figure 1).

During the last decades, ocean science has made great progress enhancing our ability to predict changes in marine ecosystems. However, we still do not fully understand the magnitude of the current problems and the best way to implement effective solutions based on scientific data (Laffoley et al., 2020). Measuring the responses of marine ecosystems to a changing ocean can be particularly challenging in remote habitats such as polar regions, the deep‐sea, or the high‐seas (Howell et al., 2021; Kennicutt et al., 2014). To that end, animal biotelemetry, or the use of electronic devices to remotely measure the physiology, behavior, or energetic status of free‐living animals (Cooke et al., 2004), has been proven useful to provide with information from these uncharted waters (Sequeira et al., 2018). Biotelemetry is a commonly applied method to investigate the movement ecology and behavior of marine fauna in relation to their environment. It has provided a scientific basis for management and conservation (Hays et al., 2019) and has significantly improved our understanding of the ecosystem functioning and dynamics (Katzner & Arlettaz, 2020; Lennox et al., 2017).

Among the different available biotelemetry techniques, Acoustic animal Tracking (AT) is the most widely used in free‐living marine organisms (Hussey et al., 2015; Matley et al., 2022). In fact, AT has traditionally produced scientific information to inform sustainable use of the oceans (e.g., Crossin et al., 2017; Friess et al., 2021; Lowerre‐Barbieri et al., 2021). In AT studies, organisms are typically equipped with transmitters that emit an ID coded acoustic signal at specific frequencies, which is then detected by arrays or gates of acoustic receivers (Heupel et al., 2006). In addition, the transmitters can incorporate specific sensors measuring environmental variables (e.g., temperature), movement, and behavioral traits (e.g., depth, acceleration, and predation events; Thorstad et al., 2013). These sensors allow to directly link the animal's behavior (3D movement and space‐use) to the surrounding environmental conditions, providing a technology to continuously monitor not only the distribution of marine biodiversity *per se* but also the changing environmental conditions (Aspillaga et al., 2017). In addition, the relatively low cost of electronic tags, their extended life span (over 10 years), the ability of arrays of receivers to generate high‐throughput accurate positional data (Nathan et al., 2022), and the possibility to monitor large numbers of individuals without the need of recapture (Aspillaga et al., 2021), make AT a promising tool to generate high‐quality scientific data to address the major targets proposed by the SDG14.

The objective of this work is to review case‐studies that show how AT and Tracking Networks of acoustic receivers (TN) can be used to provide with a roadmap for implementing and achieving the SDG14 targets by 2030 (Figure 1). In the following sections, we list target‐by‐target empirical examples and discuss how AT and TN can contribute to the task of providing high‐quality scientific outputs to design optimal strategies for the sustainable use of oceans and rebuilding marine biodiversity.

## 2 SDG14 TARGET 1—ACOUSTIC TRACKING TO MEASURE THE EFFECTS OF MARINE POLLUTION

Pollution is present in all aquatic ecosystems as a result of deliberate or accidental disturbances such as discharges from industry and agriculture, noise, artificial lighting, and dumping of solid waste (Barnes et al., 2009; Carpenter et al., 1998; Duarte et al., 2021; Larsson et al., 2007; Longcore & Rich, 2004). The first SDG14 target aims to significantly reduce marine pollution by 2030. The effects of pollutants on marine animals have been mainly studied in laboratory‐based settings (Carlsson et al., 2009; de Jong et al., 2018; Kasumyan, 2001). However, the results from such experiments can seldom be directly extrapolated to complex wild ecosystems (Bertram et al., 2022; Calisi & Bentley, 2009). Using AT, behavioral data can be directly obtained from a wild population while measuring other environmental variables such as pollution levels (Barcelo‐Serra et al., 2021). In this way, it is possible to assess the direct effects of pollution on animal behavior and welfare (Hellström et al., 2016; Huveneers et al., 2021).

Chemical water pollutants such as pesticides, fertilizers, heavy metals, and drugs have a great impact on aquatic wildlife (Kasumyan, 2001; Pyle & Ford, 2017; Sanchez et al., 2011). These pollutants, even in small concentrations can lead to biodiversity loss and pose a threat to human health via ingestion of harmful chemicals bioaccumulated in aquatic organisms. Studies using AT have assessed the effects of chemical pollution on fish mortality (Thorstad et al., 2013), habitat use (Burns et al., 2021; Crear et al., 2016; Curtis et al., 2013; Madrak et al., 2016; Moser & Lindley, 2007), and on species captured for human consumption (O'Toole et al., 2012; Taylor, van der Meulen, et al., 2018). The results of such studies show the broad applications of AT for the preservation of aquatic fauna and to ensure safe marine‐derived products for human consumption.

Chemical and oil spills as a result of industrial accidents lead to mass mortality events and long‐lasting environmental perturbations (Munilla et al., 2011; Peterson, 2003). In the event of such catastrophic incidents, having baseline information on the previous ecosystem functioning, including environmental and animal movement data, is of utmost importance for restoration plans (Bjorndal et al., 2011; Peterson, 2003). For instance, the movements of marine fauna before, during, and after oil spills have been monitored using AT, showing important short‐ and long‐term effects on physiology, behavior, and survival (Vander Zanden et al., 2016; Zięba et al., 2014). It follows that having TNs deployed in areas susceptible to pollution can provide valuable insight on animal behavioral changes in the event of chemical accidents or global crises.

**FIGURE 2:**
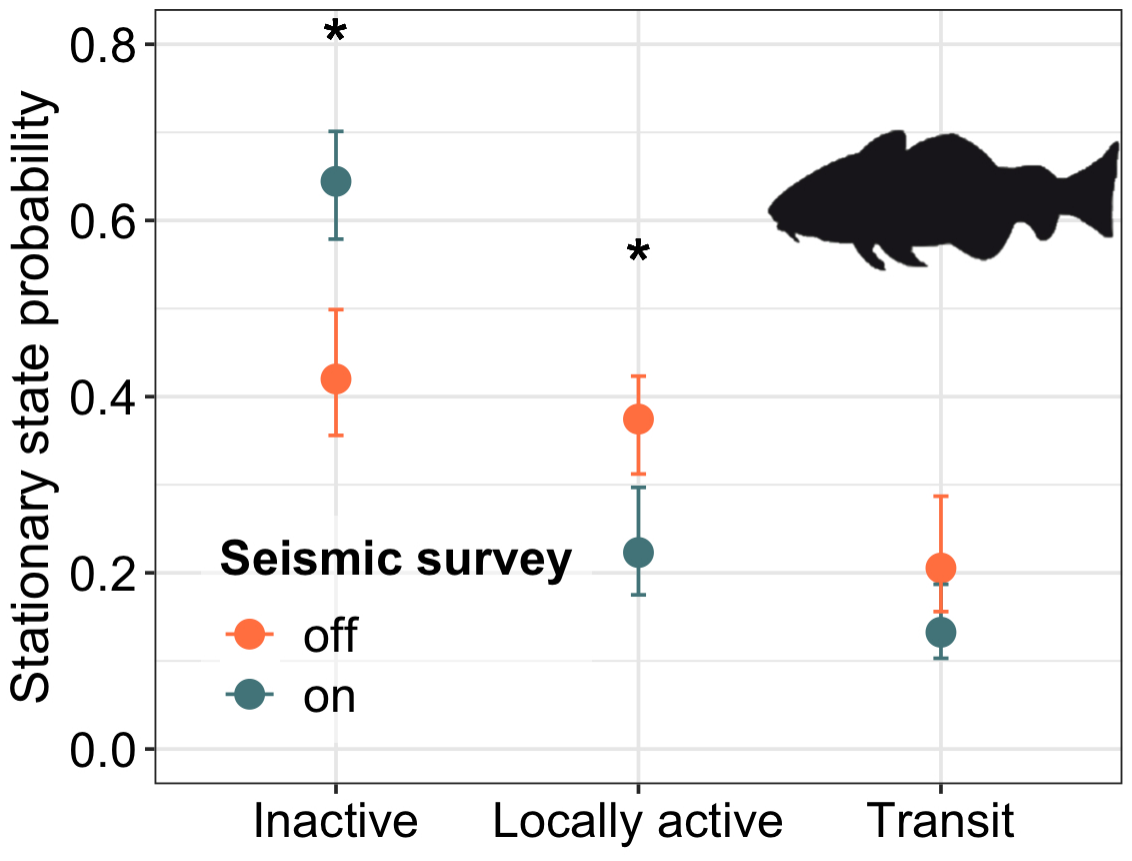
Acoustic animal Tracking (AT) can be used as a tool to measure the effects of noise pollution on fish behavior. This plot shows the results of an AT experiment on Atlantic cod *Gadus morhua* showing the effects of a seismic survey on the probability of switching between different activity states (adapted from van der Knaap et al., 2021).

Activities such as seismic surveys, mining, commercial and recreational shipping, and intense urbanization are the most important sources of anthropogenic noise and artificial lighting oceanwide (Estabrook et al., 2016; Hildebrand, 2016; Longcore & Rich, 2004). Exposure to high levels or long periods of noise has a negative impact on marine animals, resulting in hearing capacity losses, physiological, and behavioral alterations (Cox et al., 2018; Fewtrell & McCauley, 2012; Rolland et al., 2012; Simpson et al., 2016). Furthermore, the continuous presence of artificial light can compromise orientation during the hatchling dispersal of sea turtles (Salmon et al., 1995). Measuring the effects of these disturbances on aquatic animal behavior has been impaired by the lack of appropriate tools (Barcelo‐Serra et al., 2021). However, recent AT studies managed to reveal significant impacts of seismic surveys (Figure 2), shipping, and wind farm noise on fish behavioral patterns (Davidsen et al., 2019; Ivanova et al., 2020; Rider et al., 2021; Wardle et al., 2001; Winter et al., 2010) and potential effects on population survival and fisheries productivity (Bruce et al., 2018; Hubert et al., 2020; van der Knaap et al., 2021, 2022). The benefits of AT to measure the effects of light pollution is extended to other no‐fish taxa. For instance, Thums et al. (2016) and Wilson et al. (2018) also showed that, in the presence of artificial lights, turtle hatchlings alter their movement trajectories reducing hatchling survival. Given that an increase in coastal development and ocean‐based industrial activities is expected in the next decade, technologies such as AT can be used to inform strict regulations to manage the negative effects of these types of emergent pollutants (Duarte et al., 2021; Nowacek et al., 2015).

Finally, the uncontrolled dumping of solid waste is filling our oceans with debris, mainly from single‐use plastics and litter from industrial activities such as lost fishing gear (Barnes et al., 2009; Consoli et al., 2018; Li et al., 2016). Overall, over 150 million tons of plastic have now accumulated in the oceans, with 4.6–12.7 million tons added every year (Jambeck et al., 2015). There is also mounting evidence that this pollution is even reaching the deep‐sea, with surprisingly high quantities being accumulated in the sea bottom, from continental slopes to abyssal plains (Chiba et al., 2018). Given these overwhelming numbers, removing debris from the oceans seems unfeasible. However, tracking plastics with AT could help to elucidate the routes followed by debris from land into the sea (Duncan et al., 2020), including the open ocean and the deep‐sea, and thus critically inform localized management actions with a reverberating effect in the wider ocean (Bert et al., 2021).

## 3 SDG14 TARGET 2—ACOUSTIC TRACKING AS A TOOL FOR OCEAN PROTECTION AND RESTORATION

The second target of SDG14 aims to sustainably manage and protect marine and coastal ecosystems to avoid significant adverse impacts, strengthen their resilience, and act on their restoration. While marine protected areas (MPAs) are addressed by SDG14 Target 5, effective protection plans from widespread human activities and site‐specific developments that impact marine ecosystem health and biodiversity are also required. Monitoring the effects of protection and restoration of the oceans requires deep insight into the habitat use and migrations of often highly mobile aquatic animals. Such information can now be gathered using AT to inform spatial management plans, identify essential habitats, perform environmental risk assessments, or assess the impacts on marine biodiversity before and after the construction of infrastructures. For example, AT has been successfully employed to detect the behavioral impacts of shipping (Ivanova et al., 2020; Sertlek et al., 2019), dredging (Castro‐Santos et al., 2019; Wenger et al., 2017) and seismic surveys (Slabbekoorn et al., 2019; van der Knaap et al., 2021, 2022), identifying management and policy implications. Therefore, this tool should be widely promoted given the undergoing or planned expansion of blue economy marine infrastructures, namely renewable energy infrastructures and deep‐sea mining, respectively, both of which may have confirmed or suspected severe impacts (Masmitja et al., 2020).

Harvesting renewable energy from winds, currents, tides, and waves is a relatively new threat to marine ecosystems (Gill et al., 2020) and there are significant concerns on its environmental impact and sustainability (Dannheim et al., 2020). For example, wind turbines and power cables can impact marine ecosystems during construction (e.g., pile driving) and operation, introduce artificial physical infrastructure to the ocean, alter the water currents, and emit electromagnetic fields, along with elevated vessel traffic (Boehlert & Gill, 2010; Degraer et al., 2020; Gill, 2005). Using AT, we can gather pre‐development baseline spatiotemporal animal movement data. In fact, Ingram et al. (2019) suggested that AT should be a prerequisite to evaluate the impact of an offshore wind energy development to mitigate its potential negative impacts on the endangered Atlantic sturgeon, *Acipenser oxyrinchus*. Once constructed, human‐made infrastructures can also provide physical habitat for fish aggregation, influencing local biodiversity and ecosystem functioning (Halouani et al., 2020). Reubens et al. (2013, 2014) used AT and stomach content analysis to identify a seasonal preference to wind farms related to feeding but also shelter from currents and predators in a commercially important fish in the North Sea. Staines et al. (2019) showed that AT can produce high‐resolution movement data required to assess potential lethal interactions of fish with tidal turbines. Finally, Everett et al. (2020) used AT to assess the seasonal patterns of area use in northern red snapper, *Lutjanus campechanus*, to schedule the explosive removals of decommissioned platforms as to reduce impacts.

Coastal ecosystems around the world have suffered habitat loss due to urbanisation, agricultural practices, and infrastructural developments. AT can be used to gather individual‐ and population‐level knowledge on habitat use to inform habitat restoration plans and evaluate their success. For example, AT has been used to assess the functionality of artificial (Arendt et al., 2001; Eggers et al., 2015; Hindell, 2007) and restored (Espinoza et al., 2011; Farrugia et al., 2011; Jirik & Lowe, 2012) estuarine habitats. Freedman et al. (2015) established the connectivity of two discrete restored estuaries based on feeding guild, while TinHan et al. (2018) combined non‐lethal natural tracers of trophic ecology with AT to demonstrate how restored oyster reef habitat primarily benefit larger spotted seatrout, *Cynoscion nebulosus*.

Artificial reefs have been used for a long time for habitat protection and restoration purposes (Addis et al., 2013; Bombace, 1989; Clark et al., 1974; FAO, 2015). The extent to which species use artificial reefs as alternative habitats has frequently been determined by AT, bringing new information on site fidelity, home range, habitat use, and diel migration in fish species (Abecasis, Afonso, et al., 2013; Abecasis, Bentes, et al., 2013; D'Anna et al., 2011; Kristensen et al., 2017; Özgül et al., 2015; Piraino & Szedlmayer, 2014; Reynolds et al., 2010; Smith et al., 1999; Taylor, Becker, & Lowry, 2018; Topping & Szedlmayer, 2011). Additionally, AT has been employed to study connectivity and behavioral variation between species using natural and artificial reefs (Abecasis, Afonso, et al., 2013; Abecasis, Bentes, et al., 2013; Getz & Kline, 2019; Koeck et al., 2013; Logan & Lowe, 2018), residency patterns (Keller et al., 2017), interactions between species (Dahl & Patterson, 2020), and exploitation dynamics (Pioch et al., 2011). AT data on exploited reef fishes have also been used to define spatial fishing restrictions and increase fishing efficiency (e.g., Özgül et al., 2019; Topping & Szedlmayer, 2011).

Fish stock enhancement and re‐introduction are other important management and restoration measures in response to local stock depletion. For that purpose, understanding post‐release dynamics is key to maximize the effectiveness of stocking programmes (Taylor et al., 2017). Thus, the survival, site fidelity vs. emigration dynamics, and habitat selection of stocked (released) fishes have been studied using AT to measure performance of release locations (Pursche et al., 2014), stocking density (Taylor et al., 2013), and shelter acclimation (Kawabata et al., 2011). Captive bred fish can behave unnaturally in the wild due to genetic differences (local adaptation of stocks), domestication (rearing environments influencing development and learning), and acclimation to the new environment. Consequently, stocked fish movements have frequently been compared with wild fish movements using AT such as for seaward migrating anadromous salmonids (Aarestrup et al., 2014; Chittenden et al., 2008; Flávio et al., 2019; Urke et al., 2013) and marine fish (Kawabata et al., 2007; Parrish et al., 2015), including sharks (Lee et al., 2015).

## 4 SDG14 TARGET 3—ACOUSTIC TRACKING TO MONITOR THE EFFECTS OF CLIMATE CHANGE

It is now increasingly evident that human‐induced global change is profoundly affecting marine ecosystems (Nagelkerken & Connell, 2015; Poloczanska et al., 2013). The global ocean has absorbed ~90% of the excess heat from the climatic system and ~30% of the released CO_2_, steadily becoming warmer and more acidic (IPCC, 2019). In addition, oxygen concentrations have concomitantly decreased in coastal waters and beyond due to temperature increases and changes in the ventilation and biogeochemistry of the water masses (Andrews et al., 2013; Breitburg et al., 2018). One of the most evident impacts is the poleward shift in the distribution of many species due to global warming (Hazen et al., 2012; Montero‐Serra et al., 2015), yet comparatively there is still much ongoing discussion regarding the effects of ocean acidification on marine biodiversity (Clements et al., 2020). Despite physiological studies having demonstrated that acidification might affect their sensory system (Simpson et al., 2011) and increase larval mortality by predation (Munday et al., 2010), some reviews have not found evidence that fishes are being negatively affected by ocean acidification *per se* (Clark et al., 2020; Kroeker et al., 2013). Nevertheless, acute behavioral changes can be expected due to habitat change forced by decreases in pH (Nagelkerken et al., 2016). The reduction of oceanic dissolved oxygen is also thought to exacerbate the effects of warming and acidification by reducing the physiological tolerance ranges of fishes and other organisms (Deutsch et al., 2015; Pörtner & Knust, 2007).

All climate predictions forecast a worsening of the described conditions in the future (IPCC, 2019) and, consequently, the impacts of global change are also targeted by SDG14. Target 3 of the SDG14 aims to minimize and address the impacts of ocean acidification through enhanced scientific cooperation at all levels. Since ocean acidification is known to generate synergistic effects with warming and deoxygenation, all of them should be considered to safeguard the sustainable use of the oceans and marine resources in the future. Biologging technologies, such as AT, are a key tool to upscale physiological and behavioral studies in the wild (Cooke et al., 2016; Hellström et al., 2016). By using transmitters equipped with sensors (e.g., depth, temperature, acceleration) in combination with continuous environmental data monitoring, AT can be used to explore the behavioral responses of animals to fluctuating environments. These studies provide complementary information to physiological studies in the laboratory on the real impact of environmental changes in an integrative manner.

The most common examples of using AT to unveil the effects of environmental conditions on aquatic animal behavior are thermal preference studies in environments with sharp thermal gradients, such as seasonal thermoclines. These studies allowed to describe a regional or vertical preference for warm waters in many predators, from yellowfin tuna *Thunnus albacares* (Block et al., 1997) to common dentex *Dentex dentex* (Aspillaga et al., 2017), while other coastal species have shown preferences for colder waters, such as Atlantic cod *Gadus morhua* (Freitas et al., 2016), brown trout *Salmo trutta* (Kristensen et al., 2018), or dogfish *Scyliorhinus canicula* (Sims et al., 2006). The few studies using AT to look at the temperature envelopes of deep‐sea fishes have shown that in their natal regions, they typically restrict their habitat use to the cold waters below the thermocline, whether they are sedentary residents like rockfishes *Sebastes* (Starr et al., 2002) or perform daily vertical migrations of hundreds of meters such as the blackspot seabream *Pagellus bogaraveo* on seamounts of the mid‐Atlantic ridge (Afonso et al., 2012). Similarly, AT has been used to study he effect of oxygen concentration in space use patterns of aquatic organisms. Itakura et al. (2021) described a wide thermal tolerance for the stripped seabass *Morone saxatilis*, that actively avoided both high temperatures and bottom hypoxic waters during summer. Similarly, hypoxic upwelling events caused a 33% reduction in the home range size of the copper rockfish *Sebastes caurinus* (Rankin et al., 2013). All these studies provided key baseline information on the optimal environmental conditions of the studied species, which is highly useful to understand and predict their present and future population trends.

To date, there are no studies directly relating animal behavior and ocean acidification, primarily due to the difficulty that entails conducting continuous pH measurements in the natural environment at relevant scales and the current lack of strong evidences of long‐term acidification effects on fishes. However, acute pH gradients, for example, at natural CO_2_ vents, are known to generate strong changes in the local benthic communities (e.g., Hall‐Spencer et al., 2008; Linares et al., 2015). These areas consist of ideal natural laboratories where the direct and indirect effects (i.e., via habitat shifts) of ocean acidification on the activity, foraging, or space use patterns of species could be studied using AT, especially involving susceptible mobile species such as crustaceans with calcified shells. The fact that these are typically located at deep‐sea, remote environments makes it challenging yet holding promise for much discovery, being the use of autonomous vehicles to track tagged species in deep‐water constitutes to be a promising advance (Masmitja et al., 2020).

## 5 SDG14 TARGET 4—ACHIEVING SUSTAINABLE GLOBAL FISHERIES THROUGH ACOUSTIC TRACKING

Humans have exploited marine animals by way of fishing since the origin of our species (Walters & Martell, 2005). Because marine biodiversity features high in our society's demand for food and recreation, many fish stocks have been overexploited (FAO, 2021). However, many assessed stocks have shown signs of recovery and sustainable exploitation in response to the implementation of proper management regimes (Hilborn et al., 2021). In fact, marine‐derived products could feed more than 10 billion people by 2050 contingent to policy reforms, technological innovations, and the societal growing demands toward sustainable exploitation (Costello et al., 2020). Target 4 of the SDG14 aims to effectively regulate exploitation, end overfishing, and implement science‐based management plans to restore marine fish stocks at least to maximum sustainable yield (MSY) levels in the shortest feasible time.

**FIGURE 3:**
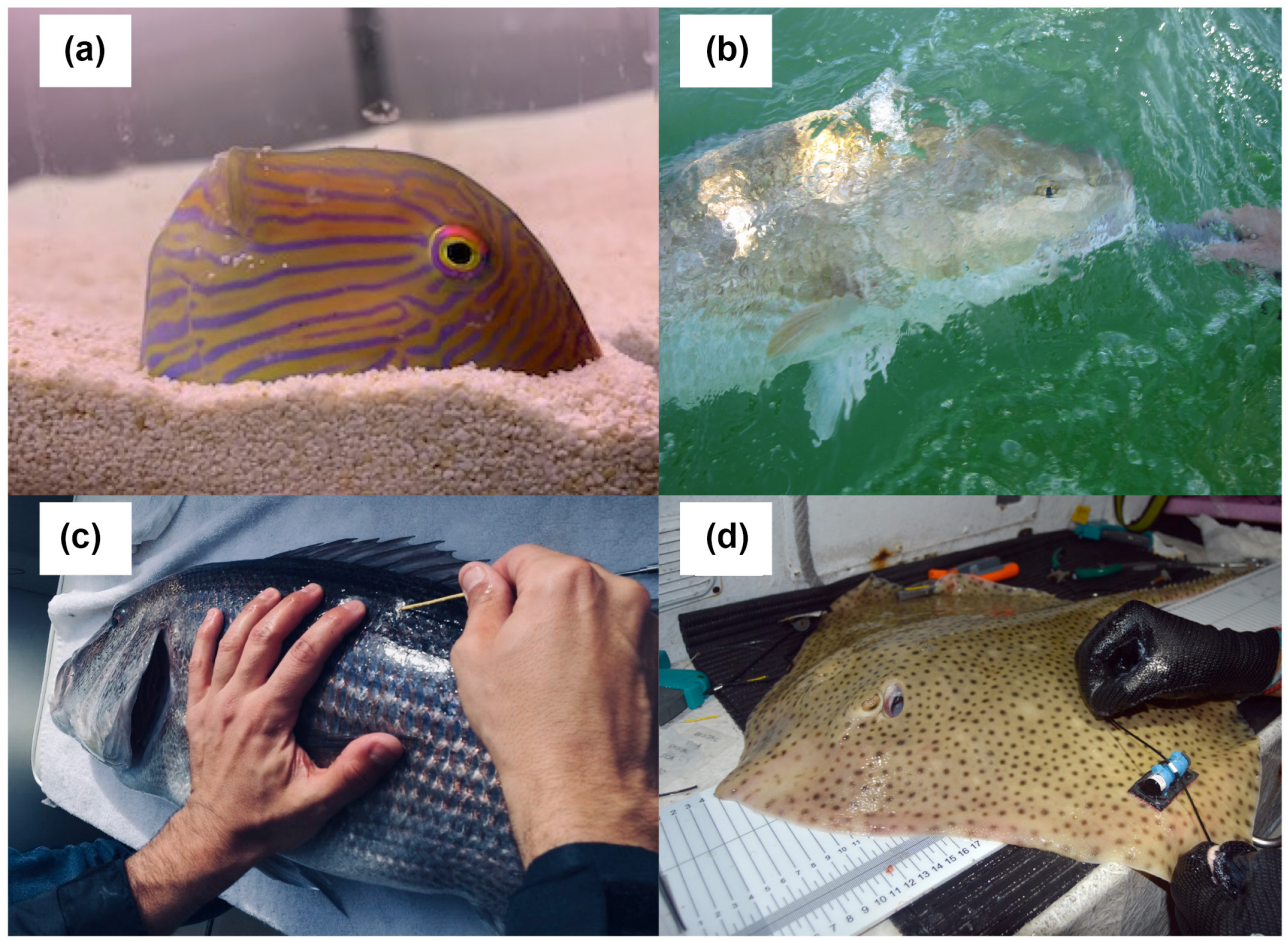
Examples of Acoustic animal Tracking (AT) as tool for the sustainable development of the world marine fisheries. (a) The pearly razorfish, *Xyrichtys novacula*: an array of acoustic receivers has revealed that movement variability plays a key role when estimating true abundance from catches (Alós et al., 2019). (b) The red drum, *Sciaenops ocellatus*: AT have been used to estimate its instantaneous monthly and annual fishing mortality, a key parameter for fisheries management, in two coastal Alabama rivers (Nelson & Powers, 2020). (c) The common dentex, *Dentex dentex*: a combination of AT and data‐driven modelling approaches provided a tool to identify aggregation areas that make this species highly vulnerable to fishing (Aspillaga et al., 2017). (d) The blonde ray, *Raja brachyura*: a fixed array of acoustic receivers revealed a 100% survival after being caught by a trammel net and discarded by small‐scale fishing boats (Alonso‐Fernández et al., 2021).

There are four major aspects underpinning sustainability in fisheries management where AT can contribute by means of high‐quality scientific data (Crossin et al., 2017; Lowerre‐Barbieri et al., 2019): allowing a more accurate delimitation of fish stocks, providing missing parameter estimates for complex population dynamics models (e.g., natural mortality), providing useful behavioral data on the vulnerability to fishing (e.g., spawning aggregations, catchability), and serving as a tool to monitor by‐catch (release) survival (Figure 3). Fisheries management is usually based on stock units defined as the biomass within a geographic region where the population is self‐sustaining (Walters & Martell, 2005). Delineation of population structure (i.e., stocks) is thus crucial to successfully manage fisheries. Historically, the spatial distribution of exploited species was described through fishers' and managers' knowledge, fisheries surveys, and a combination of mark–recapture data but had little consideration in classical stock assessments, especially at the individual level. In the last decades, AT has provided new evidences for a much more accurate definition of stock units for exploited animals (Hays et al., 2019; Lédée et al., 2021). For instance, the management boundaries encompassing stocks of the seasonally migrating Greenland halibut *Reinhardtius hippoglossoides* in the Canadian Arctic have been recently redefined according to the results of AT experiments performed in the deep‐water polar environment (Hussey et al., 2017).

AT can help attaining sustainable fisheries by providing better estimates of the parameters feeding population dynamic models that serve to estimate biological reference points (e.g., MSY). AT studies are providing better estimates of Spawning Stock Biomass (SSB) by incorporating aspects such as spawning site selection, spawning frequency, and reproductive timing (Lowerre‐Barbieri et al., 2017). AT can also directly estimate the different sources of individual mortality (natural and fishing mortality) over the years by directly measuring its variability, which cannot be done otherwise using indirect methods (Block et al., 2019; Friedl et al., 2013). For instance, Heupel and Simpfendorfer (2002) found that estimates of total mortality in juvenile blacktip reef sharks *Carcharhinus limbatus* obtained from acoustic telemetry were considerably higher than those based on a constant lifetime mortality value generated by indirect life‐history based methods. Crucially, AT renders population dynamics spatially explicit as it measures different aspects of the spatiotemporal distribution of individuals. Besides the ability to potentially describe fish stocks, AT can be used to estimate other spatiotemporal behaviors like emigration (Scheffel et al., 2020) or spatial vulnerability (Alós et al., 2012). For instance, Hightower et al. (2001) provided the first estimates of emigration (which allowed to estimate natural and fishing mortality) of males and females in a lake population of striped seabass using active AT to relocate tagged fish. Another relevant fisheries parameter that can benefit from AT is the catchability coefficient. Catchability represents the efficiency of harvesting, constituting a key link among fishers and fish stocks (Arreguín‐Sánchez, 1996). Alós et al. (2019) used AT and underwater cameras to unveil spatial behavioral types (SBT) that lead to CPUE inevitably declining faster than N (hyperdepletion) compared with a model lacking SBT, demonstrating that catchability coefficients obtained from AT may notably improve stocks assessments by providing a more reliable CPUE‐N relationship.

Sustainable exploitation may be affected by selective harvesting and its associated phenotypic change (Jørgensen et al., 2007). There is substantial evidence in the context of commercial fisheries that intensive and size‐selective harvesting selects for “fast life‐histories” (Heino et al., 2015). Movement and behavioral traits have been recently suggested to be under strong selection in fisheries either due to direct selection acting on such traits or to indirect selection emerging from correlation with life‐history (Arlinghaus et al., 2017). Hence, individual heterogeneity in relation to expressed behavioral traits such as space use, refuge seeking, energy acquisition (e.g., swimming activity), or aggression should play a major role in the catch vulnerability of fish, majorly affecting MSY (Alós et al., 2012). Several authors have demonstrated empirically this hypothesis using AT. For instance, Alós et al. (2016) demonstrated that high exploitation rates favoured individuals with small home ranges and low exploration rates, while Olsen et al. (2012) demonstrated that individuals of Atlantic cod that perform larger diel vertical migrations are more vulnerable to fishing. AT can therefore produce novel insights into the role of behavior on vulnerability and selection of individuals, and contribute to improve population dynamics models by providing better trait‐based mortality estimates.

In addition to stock and population dynamics assessment, AT can contribute to improve the management of by‐catch, that is, the incidental capture of non‐target marine animals within the objectives of the SDG14. Many fish, turtles, sharks, and birds are accidentally captured by commercial fisheries and released every year (Lewison et al., 2004). AT can provide survival estimates of discarded individuals and promote better practices to maximize survival. For example, Alonso‐Fernández et al. (2021) used a fixed AT array and recapture data to estimate short and long‐term survival of a community of coastal elasmobranchs after being captured by long‐lines, obtaining survival rates ranging from 70% to 66% in thornback ray *Raja clavata*, 100% in blonde ray *Raja brachyura* and undulate ray *Raja undulata*, and 100%–92% in dogfish. For undulate ray, the survival rate was reduced to 49% in bottom trawl fisheries using the same assessment methodology (Morfin et al., 2019). AT also demonstrated that tiger sharks *Galeocerdo cuvier* caught alive in long‐line gear experience negligible post‐release mortality only if adequately handled (Afonso & Hazin, 2014). Furthermore, the increasing participation in recreational catch‐and‐release angling generates large numbers of voluntary fish releases around the globe (Arlinghaus et al., 2019). The fate of released individuals has received substantial scientific interest and several studies suggest high survival rates (Arlinghaus et al., 2007), and several studies have demonstrated the potential of AT to determine post‐release factors to design plans to maximize survival (Donaldson et al., 2008). Ferter et al. (2015) found that Atlantic cod survived a catch and release event and did not show any behavioral changes. In a similar approach combining AT and satellite tracking, Ferter et al. (2017) demonstrated that Atlantic halibut, *Hippoglossus hippoglossus*, is resilient to catch‐and‐release angling. AT has also been used to assess the post‐release mortality due to predation in bonefish *Albula* spp. in both the Seychelles (Moxham et al., 2019) and the Bahamas (Danylchuk et al., 2007), and has demonstrated that that the stress of capture and release did not affect spawning aggregation of common snook, *Centropomus undecimalis* (Lowerre‐Barbieri et al., 2003). Curtis et al. (2015) used an array of acoustic receivers to assess the effect of capture depth in the survival of northern red snapper demonstrating a higher survival at cooler temperatures and shallower depths, and similar survival through venting the swim bladder prior to release. Finally, the survival of a range of deep‐sea fishes and sharks was assessed using AT in the Azores, also revealing the importance of good handling and release practices (O'Neill et al., 2019). Therefore, AT has contributed to demonstrate that catch‐and‐release may be an effective management strategy to reduce fishing‐induced mortality and hence promote sustainable exploitation in marine fisheries.

## 6 SDG14 TARGET 5—DELINEATING MARINE PROTECTED AREAS AND IDENTIFICATION OF ESSENTIAL HABITATS USING ACOUSTIC ANIMAL TRACKING

One of the measures to protect, preserve, and restore marine species and ecosystems that has received more attention and support from the scientific community over the last thirty years is the implementation of MPAs (e.g., Allison et al., 1998; Costanza et al., 1998; Pauly et al., 2002; Roberts et al., 2005). In fact, under the auspices of the United Nations SDG14 the target was to fully protect at least 10% of coastal and marine areas by 2020. Yet, only 7.65% of MPA coverage has been reached so far (UNEP‐WCMC and IUCN, 2021). While there is widespread recognition of the potential of MPAs to achieve conservation and fisheries management goals (e.g., Claudet et al., 2008; Goñi et al., 2010) and to buffer the effects of climatic change (Roberts et al., 2017), the proper design and functioning of MPAs is frequently impaired by the many knowledge gaps about key ecological aspects. Despite several studies on best practices for the design and management of MPAs, in most cases their implementation does not take in consideration local empirical data or spatially explicit models (e.g., Botsford et al., 2003; Grafton & Kompas, 2005; Grüss et al., 2011; McCook et al., 2010; Schmiing et al., 2009).

The advances in AT technology allowed the use of acoustic transmitters in increasingly smaller individuals and for longer periods of time, allowing scientists to obtain long term data on the individual movement patterns for many marine species, including the earlier life stages (Shillinger et al., 2012). The information provided by such AT studies has several relevant uses for MPA design and management. Many studies stress the importance of spatial information namely home range areas, site fidelity, and movement patterns for the adequate design and management of MPAs (Abecasis et al., 2014b; Costello et al., 2010; Grüss et al., 2011; Le Quesne & Codling, 2009). By estimating species home range areas AT provides pertinent information regarding the minimum size of MPAs to provide adequate protection (Green et al., 2015), and combining multispecies home range and distribution models aids assessment of multispecific MPA effectiveness (Abecasis et al., 2014b). AT can also provide information on movement barriers, which is an important aspect to consider for MPA networks design since it allows the identification of natural barriers that can therefore be used as MPA boundaries. The identification of a species' preference in habitat use can point to priority habitats for protection, from coastal waters to seamounts (Abecasis et al., 2014a; Afonso et al., 2012; Lea et al., 2016; McCook et al., 2010). This is particularly important for the conservation of highly mobile species given that the identification of essential fish habitats (e.g., spawning, nursery, feeding) allows the protection of aggregation locations and important areas for species conservation (Abecasis, Afonso, et al., 2013; Abecasis, Bentes, et al., 2013; Afonso et al., 2009; Schofield et al., 2013).

Additionally, AT studies can infer activity patterns (diel, seasonal, yearly) helping to identify migration/aggregation seasons, migration corridors, and connectivity distances of juveniles and adults, which are key aspects toward the correct design of well‐connected MPA networks (Martín et al., 2020). Although most aquatic (reef) species have their largest dispersal during the propagule phase, understanding juvenile and adult connectivity is important to fully comprehend spillover effects and thus maximize the benefits of MPA networks. Combining AT with abundance and biomass data (obtained via experimental fishing or underwater visual census) is an efficient tool to monitor the effects and assess the efficiency of MPAs providing relevant information for adaptive management (e.g., Abecasis et al., 2014a, 2014b, 2015; Lea et al., 2016; Villegas‐Ríos et al., 2021).

The recent development of spatially explicit management and ecosystem models that incorporate home range areas is also relevant even though their effective application in MPA management is still lacking (Evans et al., 2013). In addition, the use of conservation planning software, such as Marxan and Zonation (Ball et al., 2009; Moilanen & Kujala, 2008), has made the task of designing MPAs more systematic, based on different information layers (geographical, biological, socio‐economical), supported by empirical information and therefore more easily accepted by stakeholders. Information on species distribution and home range areas generated by AT can and should be used as data layers within conservation planning software as these provide highly important information, especially regarding key species and habitat protection.

## 7 SDG14 TARGET 6—PREVENTING ILLEGAL, UNREPORTED, AND UNREGULATED FISHING USING ACOUSTIC TRACKING

Illegal, unreported, and unregulated (IUU) fishing involves fishing activities that do not respect rules adopted at either national or international level contributing to unsustainable fishing practices (Pitcher et al., 2002; Sumaila et al., 2006). Combating IUU fishing is a top SDG14 priority as a mean toward a rapid and lasting recovery of fisheries (Brashares et al., 2004). In an attempt to fight IUU fishing, large sums of money are invested on the monitoring, control, and surveillance of fisheries without reaching, in most cases, the desired results (Bergh & Davies, 2002). For instance, in West Africa (one of the regions most affected by IUU fishing in the world) only ~0.5% of the economic benefits generated by IUU fishing (estimated at 2.3 billion $ per year) are recovered (Doumbouya et al., 2017). Although satellite‐based surveillance provides with a cost‐saving alternative to monitoring, control, and surveillance tools (i.e., the Vessel Monitoring Systems and the Automatic Identification System, Bruce et al., 2018; Kroodsma et al., 2018; McCauley et al., 2016; Watson & Haynie, 2016), fishing vessels can switch off or manipulate these systems to hide their identity and location (Kontopoulos et al., 2020; Long et al., 2020).

**FIGURE 4:**
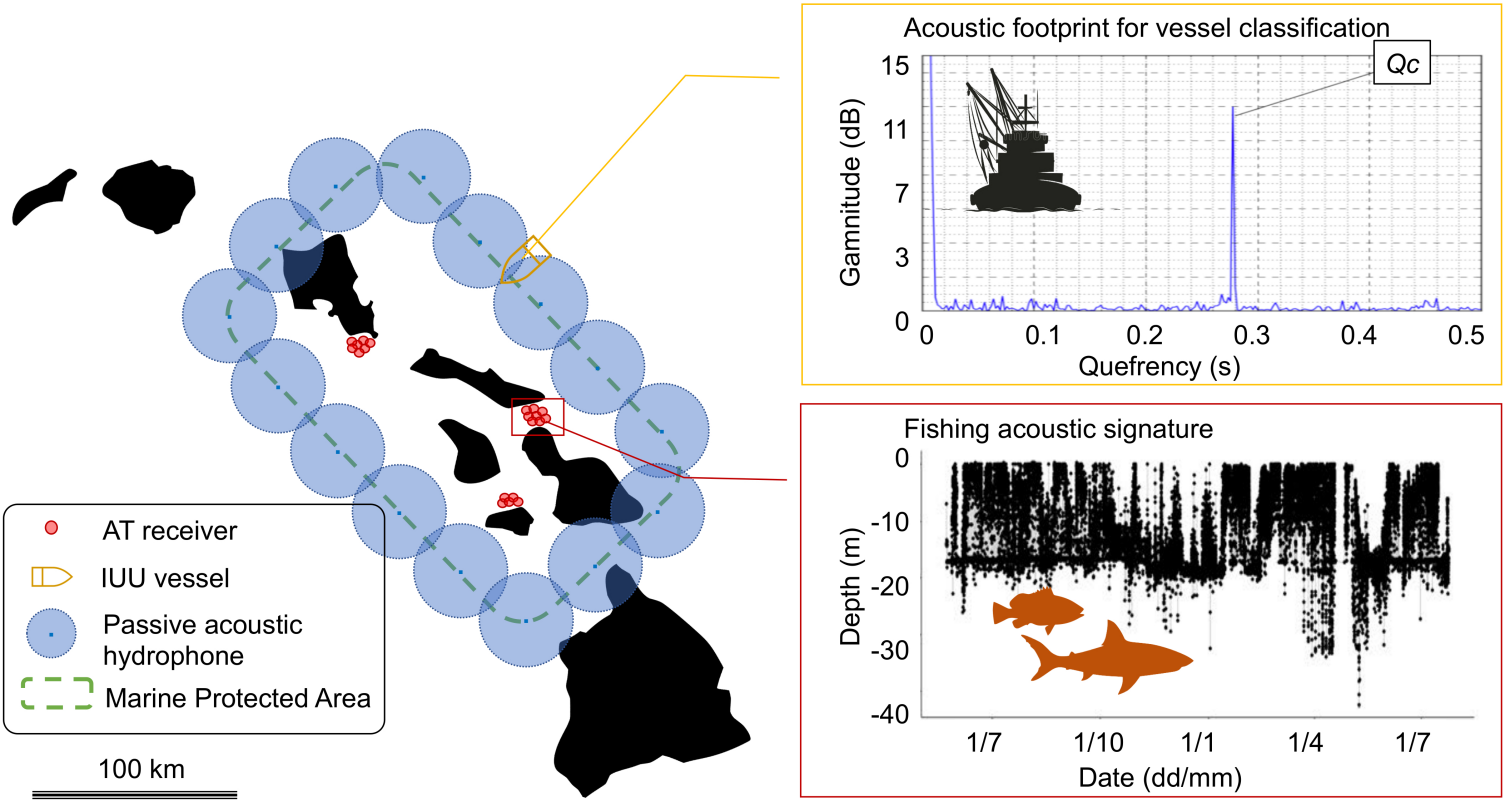
Schematic of a potential Integral Acoustic Surveillance System combining both monitoring of species and vessels at a prototypical tropical archipelago, where a marine protected area has been recently declared. Illegal, unreported, and unregulated vessels' intrusions would be detected by a passive acoustic hydrophones array (blue points, the blue shadow denotes a conservative detection range of 12 km; Salloum et al., 2018) deployed along MPA boundaries at the sea bottom with a surface antenna/satellite communication buoy to transmit illegal activity in nearly real‐time. Top yellow square details the potential acoustic footprint which would be detected and classified as a big vessel (graph extracted from Pollara et al., 2017). The peak related to engine cylinder rates are labelled with *Qc*. Several coral reefs are monitored by a dense receiver array with overlapping detection ranges (small red points with a ~1 km detection range highlighted by the red shadow). One of these dense arrays would detect the illegal harvest of target species (e.g., groupers or sharks). Red square shows a fishing acoustic signature (the first fate above‐described) detected by receivers (figure modified from Villegas‐Ríos et al., 2020).

AT provides a very useful and promising alternative tool to detect and fight IUU fishing through the monitoring of the fate of aquatic (fished) animals. Indeed, a wide array of studies demonstrated how mortality can be detected from acoustic detection patterns of tagged individuals (Heupel & Simpfendorfer, 2002; Hightower et al., 2001; Olsen et al., 2012; Pollock et al., 2004; Topping & Szedlmayer, 2013). Importantly, the added capability of AT to discriminate between fishing *vs*. natural mortality events represents an important step toward detecting IUU fishing events (Pine et al., 2003; Villegas‐Ríos et al., 2020). According to Villegas‐Ríos et al. (2020), fishing mortality can be inferred from specific detection patterns (red square in Figure 4). As an example, unusual sudden drops in activity amongst the acoustically tagged grey reef shark *Carcharhinus amblyrhynchos* and silvertip sharks *Carcharhinus albimarginatus* that commonly remain within the range of detection arrays for long periods (Barnett et al., 2012; Espinoza et al., 2015) was key to corroborate illegal operations conducted by suspicious vessels around protected coral reefs (Tickler et al., 2019).

To detect IUU activities using AT, the area intended to be protected needs to be monitored via a denser receiver array with overlapping detection ranges, and the target species needs to show high site fidelity or home range within the array (Tickler et al., 2019; Villegas‐Ríos et al., 2020). As a complementary tool, an AT monitoring system would also have the potential to detect IUU fishing via the detection of suspicious vessels using autonomous passive noise recorders (Figure 4). Such technologies, including the hydrophone Buoy (Stolkin et al., 2006) or the simplest and low‐cost Portable Noise Recorder System (Salloum et al., 2018), are able to detect, track, and classify vessels based on their noise signature (Fillinger et al., 2011; Simard et al., 2016; Pollara et al., 2017; yellow square in Figure 4). Such harmonic footprint is modulated by mechanical characteristics of the vessels (e.g., engine size, propellers cavitation; Kudryavtsev et al., 2003; Pollara et al., 2017), and as a result, a noise‐based classification of vessels can be conducted (Santos‐Domínguez et al., 2016). Moreover, the passive nature of AT and autonomous noise recorders provide a low‐cost monitoring strategy not only economically but also logistically, since these systems can cover the surveillance of several kilometres (detection distance could reach ~24 km in the open sea, Salloum et al., 2018). AT schemes are also effective in a way that do not require the buy‐in and action of multiple stakeholders, could transmit the information in real‐time, and are particularly effective in situations where silent, undetectable monitoring of IUU activity is required. It is thus reasonable to extend the application of this passive acoustic modality to the detection of vessels involved in IUU activities in combination with active AT of marine biodiversity (Salloum et al., 2018 and Figure 4).

## 8 SDG14 TARGET 7—DEVELOPMENT OF SMALL ISLAND NATIONS AND DEVELOPING COUNTRIES

Small Island Nations have strong ties to the ocean and are key stakeholders in blue growth agendas. SDG14 Target 7 focuses on increasing the economic benefits of Small Island Developing States (SIDS) and least developed countries by the sustainable use of marine resources, including through sustainable management of fisheries, aquaculture, and tourism by 2030. The anthropological history of these regions is rich in Indigenous settlements, culture, and socio‐political systems for autonomous governance that have largely been eroded by European settlement (Filous, Lennox, et al., 2020). According to the UN, the Pacific SIDS' economic zones (EEZs) cover 40 million km^2^ of the ocean and are rich in aquatic resources, including a large share of the global tuna stocks.

EEZs around small islands provide potential for both sustainable local food and economic export to international markets. Fisheries resources in these areas include coastal and reef fishes such as parrotfishes, trevallies, snappers, groupers, and triggerfishes among others (Filous et al., 2019), as well as large pelagic migrants, especially tunas and billfishes (Christ et al., 2020). Overfishing of local species is of serious concern and several important species are considered to be at risk (Cinner & McClanahan, 2006; Sumaila et al., 2013). For pelagic species that wander entire ocean basins, fisheries are confronted by jurisdictional challenges as fish pass through EEZs ephemerally before transiting into international waters where they are vulnerable to high seas fisheries.

**FIGURE 5:**
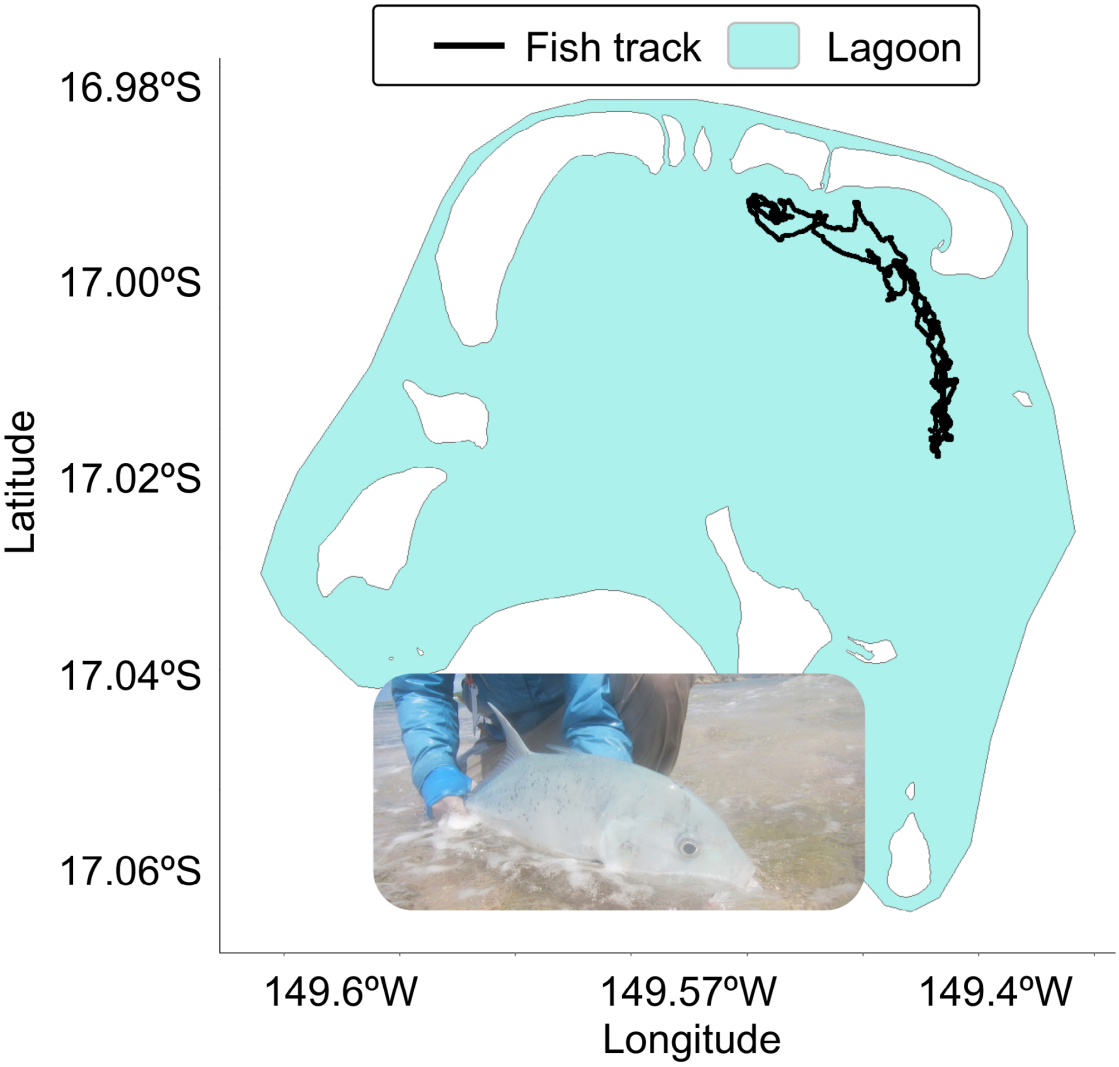
Acoustic animal Tracking (AT) can contribute, at a reasonable price, with cutting‐edge, autonomous, and easy to deploy technology that can generate scientific data to promote the economic benefits by the sustainable use of marine resources in Small Island Developing States and least developed countries. The Figure shows a track generated by an AT experiment and a picture of a giant trevally *Caranx ignobilis*, an important predator of the tropical Indo‐Pacific reefs, at Tetiaroa lagoon, French Polynesia. The results of this study provided the first detailed data on habitat use in this species and suggested MPAs as an effective tool for its conservation (Filous et al., 2019).

Monitoring and managing the resources in these coastal waters is key to inform the creation of spatial protection measures in small islands and remote areas (Chateau & Wantiez, 2007; Daly et al., 2020). Coastal arrays of AT receivers along sand flats, seagrass meadows, coral reefs, tidal creeks, and island slopes including nearby seamount summits can facilitate long‐term population monitoring to identify habitat selection, migratory corridors, spawning sites, and drivers of movement on key reef species, including the deeper demersal fishes and sharks (Afonso et al., 2012; Danylchuk et al., 2011; Filous, Lennox, et al., 2020; Figure 5). Pelagic, highly migratory species are more challenging to track, but marine buoys including platforms of opportunity such as aquatic floating drones, gliders equipped with acoustic receivers (Haulsee et al., 2015) or fish aggregating devices can be instrumental to investigate how species such as tuna associate with these devices and determine their use and vulnerability at being captured, among others research questions (Filous, Friedlander, et al., 2020). AT in pelagic zones and key aggregating sites (e.g., seamount summits) can be paired with other tracking tools (e.g., FastLoc GPS, SPOT, and PSAT tags) to generate multiscale information on animals that spend little time around the receivers.

In the context of SDG14, AT has a vast potential for action and evidence‐based management. There are a myriad of examples of AT guiding design and implementation of protected areas, which meaningfully contributes to protection and enhancement of local biodiversity. Tagging and tracking species provides residency indexes within and beyond protected areas to determine which species are protected and for how long (Daly et al., 2020; Marshell et al., 2011; Meyer et al., 2007). Sustainable use is also advanced via local and traditional fisheries management or regulatory governance. Filous, Lennox, et al. (2020) studied short jaw bonefish *Albula glossodonta* in Anaa, French Polynesia and found seasonal trends in migration of females that informed local adoption of fishing closures, which have been promising for stock rebuilding in the first few years (Filous et al., 2019; Filous, Lennox, et al., 2020). The use of AT will continue to focus on coastal species but emergence of TN and the use of platforms of opportunity may allow larger pelagic animals to increasingly be detected to inform local small‐scale fishing operations targeting these species.

Small Island Nations are disproportionately threatened by human activities resulting in sea level rise, coral reef bleaching, and fisheries overexploitation, imperilling their livelihoods and food security. There is abundant Indigenous knowledge on fish and fisheries management, and researchers should conduct their studies respectfully and with a spirit of inclusivity from study design to implementation (Reid et al., 2021). Proper permissions should be sought, and researchers should work closely with stakeholders, particularly local fishers, before, after, and during AT studies. Most researchers will be foreigners in Small Island Nations and should be cognizant of historical colonial contexts to avoid past failures (Chin et al., 2019). Indigenous knowledge systems are often complementary with western science when fish capture, acoustic receiver deployments and maintenance, and data mining are conducted cooperatively with locals (Filous, Friedlander, et al., 2020). Research on local culturally and economically important resources will usually cause local conflicts because not everyone in a community will agree on the benefits and risks, and the findings may or may not ultimately contribute to changes in management modes. Nevertheless, being inclusive, communicative, and open about AT research will usually maximize its impact.

## 9 THE NEED FOR LARGE‐SCALE, COLLABORATIVE TRACKING NETWORKS ACHIEVING SDG14 TARGETS

**FIGURE 6:**
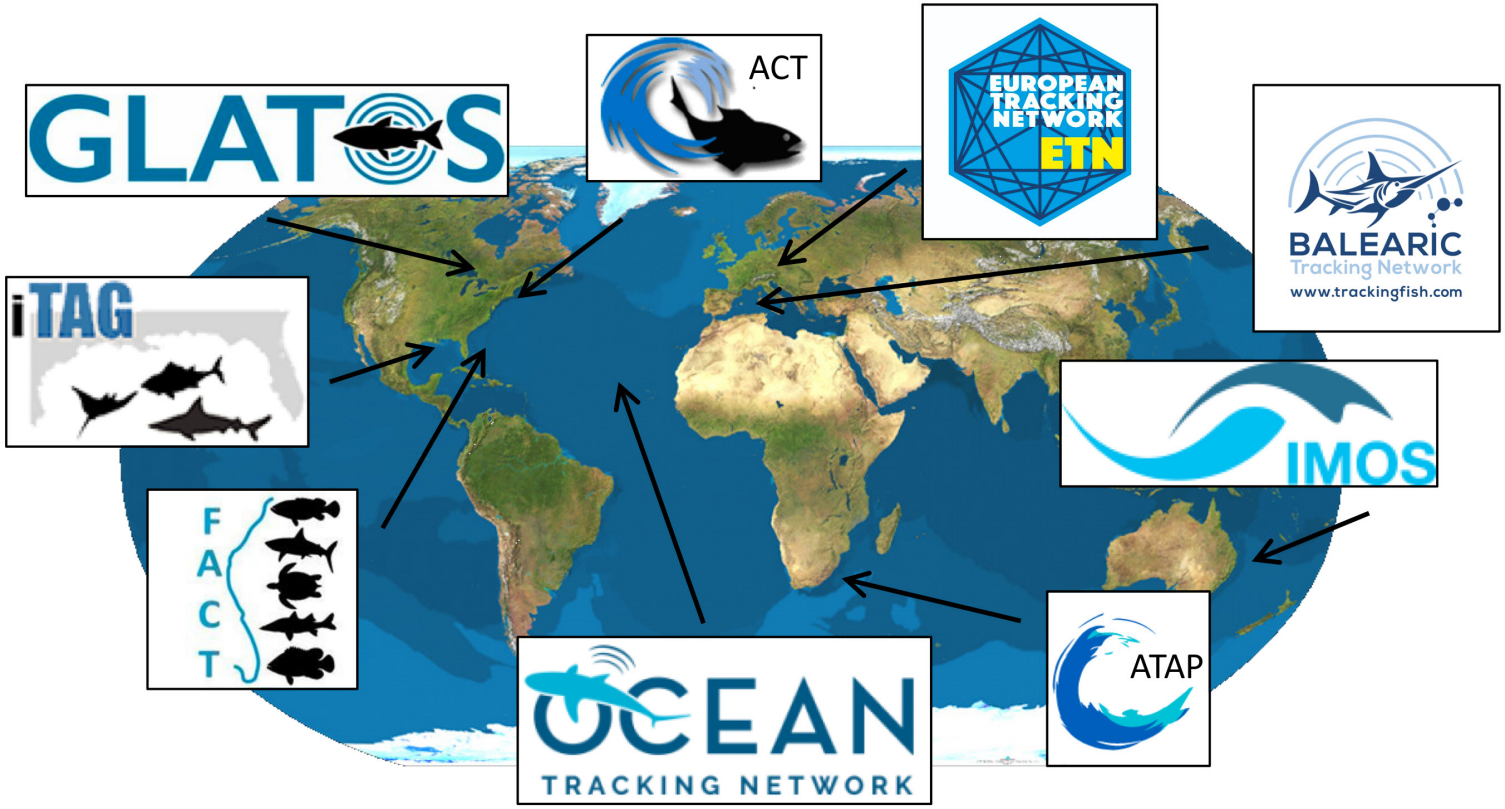
Cooperation through Tracking Networks is fundamental for a Decade of Ocean Science for Sustainable Development of Marine Biodiversity through Acoustic animal Tracking (AT). Integrated and coordinated networking of AT provides now the opportunity of tracking marine animals all around the globe in the long term (>10 years). The Figure show a selection of existing Networks: iTag—Integrated Tracking of Aquatic Animals in the Gulf of Mexico (https://itagscience.com/), IMOS—Integrated Marine Observing System (https://imos.org.au/), ACT—The Atlantic Cooperative Telemetry Network (https://www.theactnetwork.com/), FACT—Florida Atlantic Coast Telemetry Network (https://secoora.org/fact/), OTN—Ocean Tracking Network (http://www.oceantrackingnetwork.org/), GLATOS—The Great Lakes Acoustic Telemetry Observation System (https://glatos.glos.us/), ATAP—South Africa's Acoustic Tracking Array Platform (ATAP) (https://saveourseas.com/project/the‐acoustic‐tracking‐array‐platform‐atap/), ETN—European Tracking Network (http://www.europeantrackingnetwork.org/), BTN—Balearic Tarcking Network (https://trackingfish.com/).

The examples provided in previous sections have shown that AT is commonly applied to investigate the spatial ecology and behavior of aquatic species in relation to their environment. However, the SDG14 aims for sustainable development at a global level. Thus, although regional and local initiatives might play an important role to achieve the seven major Targets of SDG14 much emphasis is needed at a broader, international scale in the form of collaborative TNs. Aquatic animals do not restrict their movements to administrative borders and can migrate over extensive distances between feeding, breeding, and nursing habitats (Fujioka et al., 2018). We highlight two examples of relevant scientific outcomes that are based on TNs achieving SDG14 targets. First, Block et al. (2019) provided the first rate of instantaneous annual natural mortality, a key parameter for sustainable fisheries and MSY, for the Atlantic bluefin tuna *Thunnus thynnus* using acoustic tags and deploying acoustic receiver lines across the entrances of the Gulf of St. Lawrence in Canada. Second, to understand the implications of fish stocks being distributed across several EEZs as well as international waters, Lédée et al. (2021) used continental‐scale AT and network analysis techniques to provide novel insight on the movement of seven teleost and seven shark species. Their findings allowed to compare their results with genetic and conventional tagging studies. These two examples show how TNs provide a scientific basis for management and conservation, and can significantly improve our understanding of ecosystem functioning and dynamics (Abecasis et al., 2018; Lowerre‐Barbieri et al., 2021). This, in combination with the inherent nature of many aquatic animals to move over large distances, explains the recent need to move toward implementing large‐scale, cross‐boundary networks (Ellis et al., 2019), being the European Tracking Network (ETN, Abecasis et al., 2018), Ocean Tracking Network (OTN, Iverson et al., 2019), or Florida Atlantic Coast Telemetry working group (FACT, Young et al., 2020) some examples (Figure 6).

The leap from local studies on single species and habitats, toward international and global networks facilitating multispecies monitoring over multiple habitats and pressures creates many advantages at several levels. First, the presence of telemetry infrastructure over large geographical and temporal scales greatly enhances the potential and value of the projects and the data generated to identify key factors determining populations' health. The initially planned monitored areas and fish detections increase and research collaborations are facilitated (Ellis et al., 2019). Second, most TNs provide data management services, which improve efficiency, encourage data sharing, and facilitate the access to automated data management (Young et al., 2020). Leading to collaborations and stakeholder engagement at a broader scale, over different species and habitats (Abecasis et al., 2018; Reubens et al., 2019), resulting in an increased funding success for TNs. Third, these global networks allow for the multi‐decadal detection of unexpected and unknown movements, as well as shedding light on the somewhat restricted acoustic monitoring on long‐distance migrants.

## 10 DISCUSSION AND CONCLUSIONS

We have reviewed how AT can actively contribute to achieve most of the SDG14 Targets. Overall, the applications of AT in the study of the effects of pollution on species survival, distribution, and movement is of most importance for the preservation of aquatic fauna and ensure safe marine‐derived products for human consumption. The effects of anthropogenic sound pollution (an emergent pollutant) in marine organisms have not yet been extensively studied (Duarte et al., 2021). It is known that certain marine species are attracted or repulsed by artificial light sources constituting a type of pollution (Marangoni et al., in press). More research is needed to understand the ecological implications of such behavioral changes (Nightingale et al., 2006). AT is a promising monitoring tool that can help the development of informed managerial decisions by directly measuring the behavioral effects related to the long‐term exposure to pollutants.

AT makes possible to establish a baseline behavioral data that should be gathered prior to the deployment of large human‐infrastructures (e.g., current plan for deploying large‐scale marine wind farms across Europe). AT has been successfully used to monitor the response of marine fish to restoration‐monitoring programs, or to evaluate the effectiveness of artificial reefs (Abecasis, Afonso, et al., 2013; Abecasis, Bentes, et al., 2013; Espinoza et al., 2011). Stock enhancement and repopulation of marine biodiversity is also an important management and restoration measure in response to poor fishery performance or to compensate for stock depletion (Taylor et al., 2017). AT serves as a tool, not only to measure the survival of stocked animals, their movement, and habitat selection to identify the importance of release location but also to measure the impacts on native biodiversity (Pursche et al., 2014; Taylor et al., 2013).

The stratification of the water column has become stronger and extreme events, such as heatwaves, are now more recurrent than before (IPCC, 2019). AT has a great potential to improve our understanding on the long‐term effects of climate change on aquatic organisms by studying the effect of environmental variables on free‐ranging organisms. AT infrastructures encompassing a wider range of climatic conditions, local stressors, and flagship sentinel species, will be key to extending the behavioral and physiological studies to the scale required by the social and conservation challenges, and to achieve sustainable management international commitments (Abecasis et al., 2018).

Sustainable fisheries rely on effective, evidence‐based fisheries management actions. AT is currently an underused resource within formal fisheries management (Matley et al., 2022), where its potential to directly delineate stocks or estimate demographic parameters has yet to be fully developed (Crossin et al., 2017). AT can provide estimates of post‐release mortality allowing for the identification of ways to reduce it (e.g., through gear modifications, Bettinger et al., 2005). A central feature of the agenda and a goal of the oceans SDG14 is the protection of at least 10% of coastal and marine areas. In fact, target 5, related to MPA will dominate the SDG14 measures since protected areas are a ‘privileged’ solution in conservation, specifically in marine conservation. Targets of becoming MPAs are (relatively) SMART (Specific, Measurable, Achievable, Realistic, and Time‐bound); and the opportunity for MPA expansion is vast. As advocacy for MPAs grows around the world, it is essential that MPA scientists directly tackle the challenges of evaluating the performance of MPAs using the best available scientifically based data, and AT can demonstrate the potential both before (baseline), during, and after the protection (Dwyer et al., 2019; Martín et al., 2020).

SDG14 aims to effectively regulate harvesting and end IUU fishing and destructive fishing practices. We have shown how combining both acoustic strategies (acoustic monitoring of species and vessels) with an Integral Acoustic Surveillance strategy (Figure 4) would contribute to the task of fighting IUU fishing as a hidden and cost‐effective surveillance tool, as well as providing with a complementary technique to the monitoring, control, and surveillance strategies (Salloum et al., 2018). The use of AT will continue to focus on coastal species but emergence of TNs and the use of platforms of opportunity may allow larger pelagic animals to increasingly be detected and thus inform local small‐scale fishing operations targeting these species and specially focusing on the economic benefits to Small Island Developing States and least developed countries.

The open ocean and the deep‐sea make up for the vast majority of our oceans' surface and volume, yet we are still lagging well behind in understanding the functioning and interactions of these ecosystems. We are only now perceiving that oceanic organisms, including large vertebrates, appear to use these ecosystems tridimensionally in ways that challenge the classical scientific views (Braun et al., 2022). Understanding how these higher trophic levels depend on the massive yet threatened mesopelagic biodiversity and biomass (Martin et al., 2020), orders of magnitude higher than in coastal areas, will be key to understand how and if humanity can sustainably exploit this resource or else embark in a Pandora's box (St John et al., 2016). AT clearly has a large potential to expand its currently scarce use in deep‐sea habitats such as slopes and seamounts (e.g., Afonso et al., 2012; Hussey et al., 2017; Masmitja et al., 2020), including the least known habitats (hydrothermal vents, abyssal plains), albeit this would require a functional response from manufacturers to offer equipment that can go substantially deeper than current depth ratings (500 m).

Finally, to fully exploit AT and achieve the Targets of the SDG14, it requires a trans‐boundary collaborative effort in the form of global networks. TNs that can cover substantial volumes of the open ocean are a challenging endeavour, given the current limitations in range that AT offers. However, it remains as a promising tool especially if designed to be placed at ecological ‘hotspots’, that is, protected areas on the high seas (Maxwell et al., 2020). The ultimate goal of TNs is to leverage research that is capable of addressing social challenges, defined under the SDG, and to achieve Good Environmental Status of our oceans and seas. Decision‐making and management practices and policies should be based on sound and excellent science, which is enabled by the TNs and will directly benefit the long‐term economy and environmental status of the oceans. Achieving the trade related targets of SDG14 requires the catalysis of policies, investment, and innovations to restore the productive capacity of the oceans and increase economic benefits to developing countries, in particular Small Islands Developing States and least developed countries. Innovative AT‐based solutions that integrate best practices for harvesting, value addition in processing, and distribution can benefit greatly from opportunities offered around the concepts of ocean economy/blue economy and eco‐labelling.

## CONFLICT OF INTEREST

All authors declare no conflict of interest.

## Data Availability

The manuscript does not contain data as it is a Review of cases.
